# Consensus: a framework for evaluation of uncertain gene variants in laboratory test reporting

**DOI:** 10.1186/gm347

**Published:** 2012-05-28

**Authors:** David K Crockett, Perry G Ridge, Andrew R Wilson, Elaine Lyon, Marc S Williams, Scott P Narus, Julio C Facelli, Joyce A Mitchell

**Affiliations:** 1 University of Utah School of Medicine, Biomedical Informatics, 26 South 2000 East, Salt Lake City, UT 84112, USA; 2 ARUP Institute for Clinical and Experimental Pathology, 500 Chipeta Way, Salt Lake City, UT 84108, USA; 3 Intermountain Healthcare Clinical Genetics Institute, 324 10th Avenue, Salt Lake City, UT 84103, USA

## Abstract

Accurate interpretation of gene testing is a key component in customizing patient therapy. Where confirming evidence for a gene variant is lacking, computational prediction may be employed. A standardized framework, however, does not yet exist for quantitative evaluation of disease association for uncertain or novel gene variants in an objective manner. Here, complementary predictors for missense gene variants were incorporated into a weighted Consensus framework that includes calculated reference intervals from known disease outcomes. Data visualization for clinical reporting is also discussed.

## Background

For appropriate and effective patient treatment, relevant clinical information should be available to the clinician on demand. Accurate interpretation of gene test results, including phenotype association of gene variants, is an important component in customizing patient therapy. Recent endeavors such as the NCBI Genetic Testing Registry, MutaDATABASE, 1000 Genomes and the Human Variome Project draw attention to this growing interest in gene variant annotation and clinical interpretation in human disease [1-4]. Ongoing efforts to catalog human genome variation for many years have led to authoritative repositories of gene variants, with clear association to disease phenotype finally beginning to emerge [5-8].

Rapidly evolving technologies such as SNP chip genome-wide association studies and next-generation sequencing have lowered the cost and increased the speed of genomic analysis, yielding much larger data sets [9]. Currently, gene variants are being discovered at an unprecedented pace. One recent report found an average of 3 million variants per personal genome [10]. Unfortunately, an ever-widening gap exists between this fast growing collection of genetic variation and practical clinical interpretation due to a lack of understanding of the phenotypic consequences (if any) of any given variant. Although the number of genetic testing laboratories has remained around 600 over the past several years, recent data show that clinical testing is currently available for well over 2,200 different genes or genetic conditions [11]. As medical records increasingly incorporate genetic test information, improved decision support approaches are needed to provide clinicians with the preferred course of treatment [12,13]. Furthermore, for decision support rules to be of value, the clinical relevance of laboratory information must be well understood [14,15].

Updated recommendations have been proposed from the American College of Medical Geneticists (ACMG) on reporting and classification of sequence variants, including approaches to help determine the clinical significance of variants of uncertain significance [16]. These guidelines delineate six interpretative categories of gene sequence variation, with defined classifications outlined and the hope of a unified standard terminology in gene test reporting. For improving interpretation of unclassified genetic variants, definitions and terminology have also been recommended by the International Agency for Research on Cancer (IARC), part of the World Health Organization [17].

Despite these recommendations, however, for genetic laboratories to unify and standardize terminology and classification of gene variant test reporting, various terms such as 'deleterious', 'mutation', 'pathogenic' or 'causative of disease' are still being used [18]. In a similar vein, test results such as 'indeterminate', 'unknown', 'uncertain', 'unclassified' and 'undetermined' make it difficult to interpret the significance of a gene test result. Further compounding this issue, word modifiers such as 'likely', 'suspected', 'predicted' and 'mild', 'moderate' or 'severe' sometimes also accompany variant classification. Of this environment, one recent study perceptively noted, 'The outcome of this inconsistency for clinicians and patients in such cases is uninformative; unhelpful at best and, at worst, open to misinterpretation' [19]. In this light, the prevailing question becomes how to best help clinicians faced with decisions around gene variants of uncertain significance.

A brief review of the literature indicates that gene test reports of variants of uncertain significance range widely. One laboratory site reported that from 30% to 50% of sequence changes reported for *BRCA1 *and *BRCA2*, respectively, were reported as variants of uncertain significance [20]. Similarly, analysis of a second laboratory revealed that a physician who orders *BRCA1 *and *BRCA2 *testing had an equal likelihood (13%) of receiving an uncertain variant result as seeing a test report containing a known pathogenic mutation [21]. More recent data indicate that identification of variants of uncertain significance has continued to decline to approximately 5% of *BRCA *tests performed - a testament to the importance of maintaining and updating variant databases [22].

Another well-known example is hereditary nonpolyposis colorectal cancer syndrome, where according to the US Preventive Services Task Force and others, a clinician may expect some 13% to 31% of tests reports to say mutation of unknown significance (uncertain variant) [23,24]. An uncertain variant indicates that the risk of cancer is not fully defined and patient treatment is then based on personal and family history of cancer. Clinicians may be further frustrated when the chance of receiving a test report containing an uncertain variant is even higher for individuals from under-represented ethnic groups due to insufficient data on common polymorphisms from that population [25]. Additionally, newly identified variants from known genes present a greater challenge for interpretation of sequence-based results because they lack traditional confirming evidence of disease association [26].

Clinician frustration and obstacles to wide adoption of proposed guidelines may be two-fold. First, the lack of any quantitative metric or standardized scale for evaluation of novel or uncertain gene variants makes each difficult test result interpretation subjective to location and expertise at hand. A second and closely related challenge is the lack of an objective and standardized framework or context to make that metric meaningful. This quantitative metric and framework for evaluation become especially critical for interpretation of novel and uncertain gene variants where there is the obvious lack of existing evidence, such as family history, pedigree trios or sib pairs, confirming literature reports, bench assay biochemical evidence or colleague consensus of disease association.

In an effort to increase the transparency of providing gene variant evidence in test reporting to the clinical setting, we here present an implementation of our recently reported gene-specific predictor (Primary Sequence Amino Acid Properties (PSAAP)) into a standardized framework, in which results are systematically compared with those of other computer-based prediction methods for missense variants. Finally, with analogy to conventional laboratory testing, this Consensus model of complementary predictors also calculates gene variant 'reference intervals' using known disease outcomes. Examples of visualization are also explored for augmenting diagnostic decision making.

## Materials and methods

Several clinically curated disease sets of gene variants with known pathogenicity are publicly available at ARUP Scientific Resource for Research and Education [27]. Each database relies on both medical and molecular expertise, and uniquely displays mutation and clinical information together. All sequence variants are verified for genomic position within a given reference gene and named following standard Human Genome Variation Society (HGVS) nomenclature [28]. Archived non-synonymous substitution variants were accessed from the *RET *proto-oncogene database in January 2012 [29].

Established prediction algorithms were chosen with various and complementary methodologies, such as amino acid substitution penalties, structural disruption, sequence homology (ortholog conservation) and neural nets. Mutation prediction was then performed for known benign (*n *= 46), known pathogenic (*n *= 51) and uncertain variants (*n *= 45) using our gene-specific PSAAP algorithm, and established algorithms MutPred [30], PMut [31], PolyPhen [32] and SIFT [33] as previously described [34,35]. Prediction analysis was performed during December 2011 and January 2012 using the respective default settings for each algorithm.

Descriptive statistics such as mean, median, standard deviation, minimum and maximum were calculated for the numerical output from all five prediction algorithms. Normality of the variable distributions was assessed using the Shapiro-Wilk test, where the null hypothesis assumes that the data are normally distributed and interpreted by a *P*value greater than the chosen alpha level means a normal distribution was found. Next, for predictor variables that were found to be statistically and significantly in a non-normal distribution, Spearman correlation coefficients were calculated to evaluate correlation between predictors. To account for correlations between predictor variables, and to establish a parsimonious subset of predictors, principle components were calculated using factor analysis. Finally, the resulting significant principle components were used to develop a set of linearly independent predictor values. The weighted average of the five predictor scores was then calculated as the 'Consensus' score (Table 1). All calculations were performed using SAS software, version 9.1 (SAS Institute Inc., Cary, NC, USA).

**Table 1:** Working example of calculating the weighted Consensus score

Predictor	Prin1		Prin2		Prin3	
PSAAP	0.56		0.25		0.25	
MutPred	0.62		0.14		0.22	
PMUT	0.46		0.36		-0.06	
PolyPhen	-0.27		0.40		0.77	
1-SIFT	0.06		0.79		-0.54	
Variant^a^	PSAPP^b^	MutPred^c^	PMUT^d^	PolyPhen^e^	SIFT^f^	
Pathogenic						
*C609Y*	0.85	0.90	0.98	0.97	0.00	
Vector1 =	0.85 × 0.56	+0.90 × 0.62	+0.98 × 0.46	+0.97 × -0.27	+(1-0.00) × 0.06	= 1.283
Vector2 =	0.85 × 0.25	+0.90 × 0.14	+0.98 × 0.36	+0.97 × 0.40	+(1-0.00) × 0.79	= 1.869
Vector3 =	0.85 × 0.25	+0.90 × 0.22	+0.98 × -0.06	+0.97 × 0.77	+(1-0.00) × -0.54	= 0.559
					**Weighted sum (× 100)**	= **371.1**
Benign						
*V376A*	0.07	0.13	0.19	0.04	0.60	
Vector1 =	0.07 × 0.56	+0.13 × 0.62	+0.19 × 0.46	+0.04 × -0.27	+(1-0.60) × 0.06	= 0.220
Vector2 =	0.07 × 0.25	+0.13 × 0.14	+0.19 × 0.36	+0.04 × 0.40	+(1-0.60) × 0.79	= 0.436
Vector3 =	0.07 × 0.25	+0.13 × 0.22	+0.19 × -0.06	+0.04 × 0.77	+(1-0.60) × -0.54	= - 0.150
					**Weighted sum (× 100)**	= **50.6**

^a^RET_HUMAN (UniProt #P07949) used as reference amino acid sequence. ^b^Primary Sequence Amino Acid Properties (PSAAP) algorithm, gene-specific trained. ^c^Analyzed with default settings at [57]. ^d^Analyzed with default settings at [58]. ^e^Analyzed with default settings at [59]. ^f^Analyzed with default settings at [60].

Next, with analogy to calculating analyte reference intervals for age or gender in traditional laboratory testing, a 'reference range' of Consensus scores for *RET *gene variants with known disease outcome was calculated using EP Evaluator 8 (Data Innovations, South Burlington, VT, USA). A nonparametric reference interval was used for benign (*n *= 46) and pathogenic (*n *= 51) with 95% confidence intervals (CI) for the lower and upper bounds (Table 2). The confidence ratio of the reference interval was also calculated. Due to the reciprocal nature of the SIFT score (where a lower prediction value corresponds to more 'pathogenic'), 1-SIFT was used. All predictor scores were normalized to a scale of 0 to 100.

**Table 2:** Consensus score reference intervals for *RET *gene variants

Disease outcome	N	Lower limit value	95% CI	Upper limit value	95% CI	Confidence ratio
Benign	46	85	< 76 to 98	243	231 to > 255	> 0.09
Pathogenic	51	305	< 287 to 319	462	458 to > 470	> 0.16

**Figure 1:**
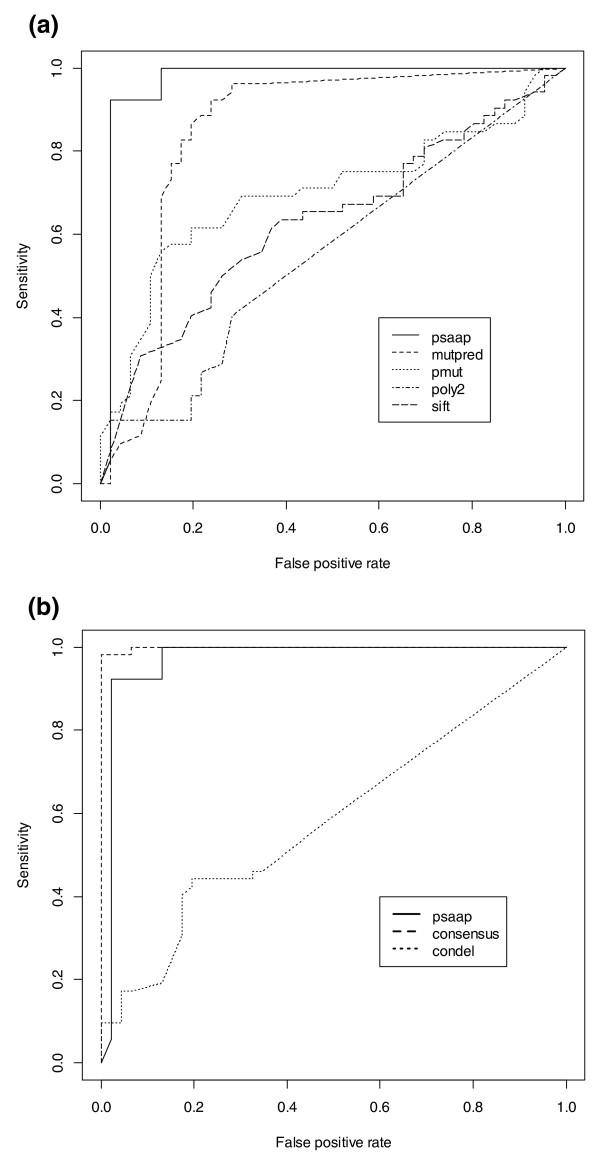
**Cross-validation for individual predictor performance**. **(a) **Calculated area under the curve (AUC) of *RET *predictor cross-validation results for individual predictor performance, including PSAAP (0.971), MutPred (0.845), PMut (0.698), PolyPhen-2 (0.555) and SIFT (0.630). **(b) **AUC results for the gene-specific predictor PSAAP (0.971), the combined predictor Consensus (0.998) and ConDel (0.587) with overcall of 'pathogenic' results for actual benign variants.

Finally, in order to better evaluate the performance of the Consensus framework, a comparison to Condel [36] and SNPs&GO [37] was performed. Further, five-fold cross-validation was implemented and performed using the Weka software package [38]. Cross-validation area under the curve (AUC) was calculated and plotted using R (v2.14.2) and the ROCR package as shown in Figure 1. We also retrospectively removed seven *RET *gene variants with known disease association (two benign, five pathogenic) from training and test sets and repeated analysis using the proposed model framework. Disease outcome predictions and Consensus scoring were evaluated for each of these variants.

Appropriate graphical summary of diagnostic information, including predictive algorithms is key for visualization and interpretation of any results generated [39]. We have loosely based the Consensus display on output from representative algorithms such as Scolioscore and FibroTest, where sample test reports are shown in Figures S1 and S2 in Additional file 1, respectively [40,41]. Finally, the use of radar (radial) plots is well documented and serves to preserve the contribution of each predictor in the weighted Consensus sum [42,43].

## Results and discussion

Prediction results (numerical output) from the five algorithms were obtained for *RET *gene variants with known disease association of benign (Table S1 in Additional file 2) and pathogenic (Table S2 in Additional file 2). Predictor results for *RET *gene variants with no reported disease association (uncertain) are summarized in Table S3 in Additional file 2. Results of correlation between predictors and significance of correlation are summarized in Table S4 in Additional file 2. Substantial correlation was seen in at least three of the five predictors (MutPred, PSAAP, and PMut). This significant correlation between variables indicates that a simple linear sum of predictors could not be used to combine the prediction scores. A weighted predictor sum (Consensus) required linear transformation of predictor outputs as determined by factors analysis.

Factor analysis was performed using principal components to determine weights of association between the five different predictors. More specifically, a set of eigenvectors was applied to weight each predictor accordingly by eigenvalues from principal components, with > 80% cumulative variance explained reached using only the first three eigenvalues. Factor analysis and cumulative percent variance explained by eigenvectors is detailed in Figure S3 in Additional file 1. PRINCOMP results and eigenvalues are summarized in Table S5 in Additional file 2.

**Figure 2:**
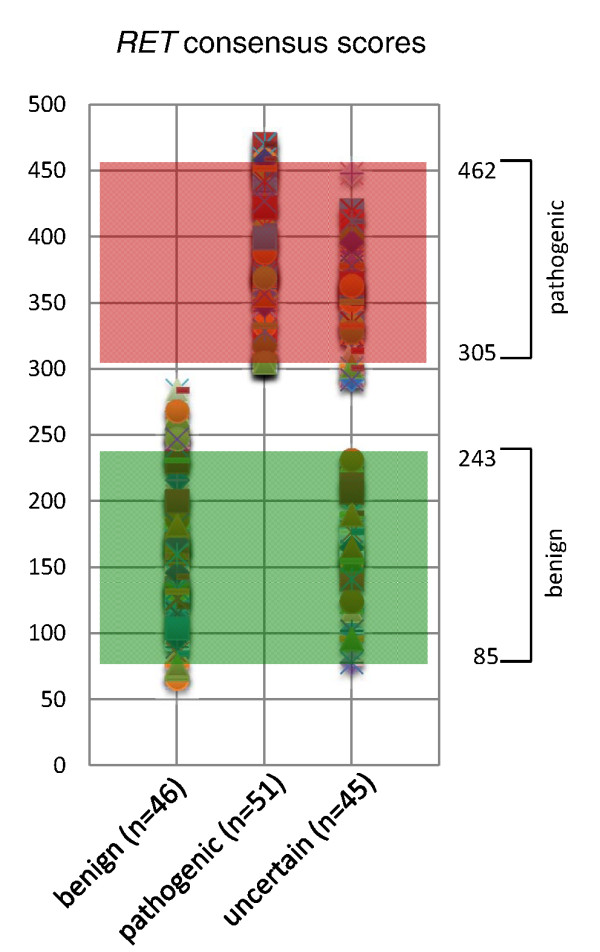
**Gene-specific reference intervals**. Scatter plot visualization of unweighted Consensus scores for *RET *gene variants, including known benign, known pathogenic disease association and gene variants of uncertain significance, demonstrating the utility of reference interval metrics for predicted benign and predicted pathogenic.

A working example of the Consensus score for both a known benign and known pathogenic *RET *gene variant is detailed in Table 1, where each predictor sum is weighted and scaled to 100. Using this same method to sum each of the five predictors for each gene variant, we then computed reference range metrics for benign and pathogenic variants for the *RET *proto-oncogene. Benign variants ranged from 85 to 243, while pathogenic variants ranged from 305 to 462. Confidence ratios for the calculated reference intervals were 0.09 and 0.16, respectively. The *RET *gene variant Consensus reference intervals are summarized using scatter plot distribution of scores for benign and pathogenic as displayed in Figure 2. Further demonstrating the utility of a reference interval metric for gene variants, the distribution of Consensus scores for prediction of *RET *uncertain gene variants shows approximate groupings into reference interval ranges also plotted in Figure 2.

In combination, the overall Consensus score may augment the rare instance that a gene-specific prediction does not outperform the existing tools. Some advantage of Consensus over existing predictors was demonstrated by performing a comparison with recently popular tools such as Condel and SNPS&GO [36,37]. The comparison demonstrated a surprisingly accurate agreement among gene variants (*n *= 121) with known pathogenic disease outcomes, where Condel showed 99.2% agreement and SNPs&GO 93.4% agreement. For variants with known benign outcomes (*n *= 67), however, Condel scored only 17.1% agreement, while SNPs&GO was slightly better with 28.6% agreement. Results for five-fold cross-validation showed acceptable reproducibility with 97.9% precision and 93.5% recall, yet a trend of overcalling disease causing predictions was readily apparent, as seen in Figure 1.

Further, to approximate the longitudinal and moving target of phenotype curation, Consensus performance was also retrospectively confirmed by removing seven *RET *gene variants with known disease association where originally they were classified as variants of uncertain significance. After excluding these seven variants from the gene-specific training set, analysis using the Consensus framework was repeated. Due to the lack of a representative variant in the training data, PSAAP only called disease association correctly for five out of seven variants. In combination, however, the Consensus score correctly predicted the sixth variant. Closer inspection showed the remaining seventh variant was a nucleotide-level 'silent' polymorphism (no amino acid change), which may have been recognized by splice effect prediction software.

**Figure 3:**
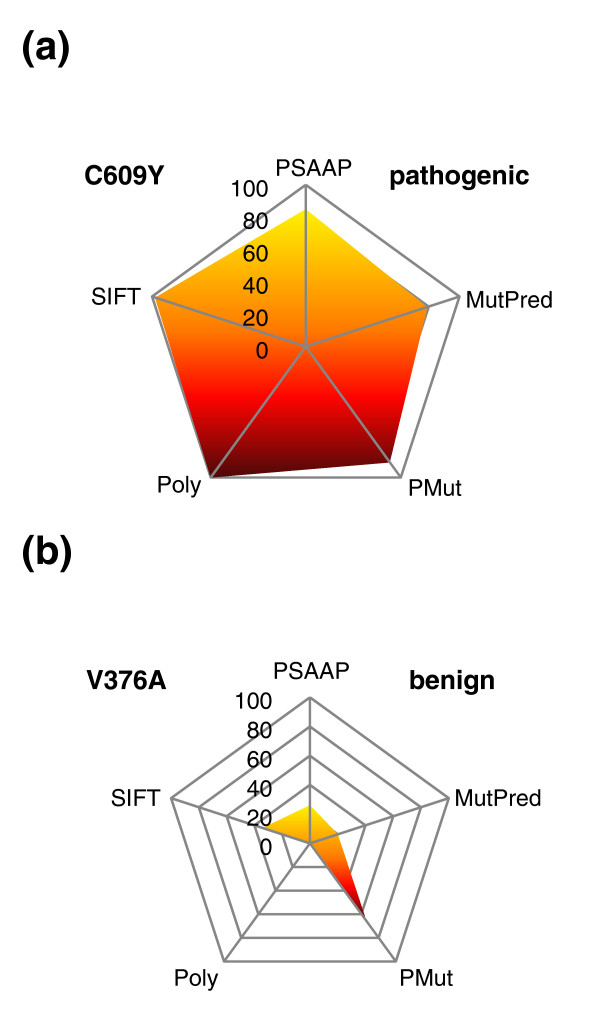
**Plotting individual predictor contribution**. Using radar plots for Consensus scoring preserves the contribution of each predictor to the total sum. **(a) **Consensus score plot of 470 (85, 90, 98, 97, 100) for the pathogenic gene variant C609Y. **(b) **Consensus output of 103 (7, 13, 19, 4, 60) for a benign variant V376A. Individual predictor scores are shown here as unweighted.

**Figure 4:**
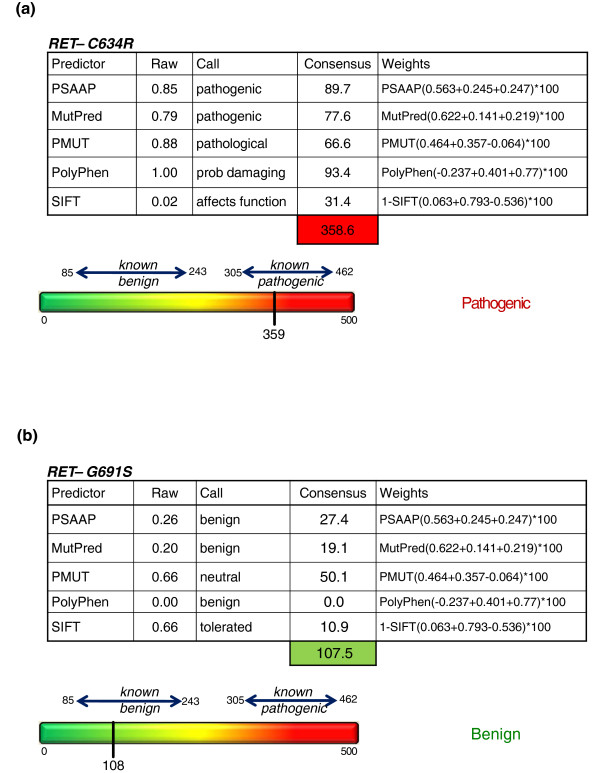
**Consensus score display**. **(a, b) **Visualization of the five-predictor Consensus model, including algorithm output, predictor calls, weighting sum and colorimetric scale for pathogenic gene variant C634R (a), scoring 359, and benign variant G691S (b) with a Consensus score of 108.

Finally, one common graphing display technique to preserve contribution of each variable (predictor) is the use of radial plots (also known as radar or spider plots). *RET *Consensus scoring results (unweighted) for the pathogenic variant C609Y and benign variant V376A are shown using radar plots in Figure 3. For augmenting clinical decision making, a more comprehensive display for Consensus scoring is shown in Figure 4, which incorporates algorithm output, predictor calls, weighted sum and colorimetric scale.

Currently, there is no widely accepted computational predictor in clinical use for evaluating uncertain gene variants. Furthermore, a lack of standardized framework and quantitative metric for evaluation of disease association of novel and uncertain variants remains an obstacle to widespread implementation of proposed guidelines and definitions of gene test reporting. The analogy of conventional laboratory analyte testing with established cutoffs and reference intervals may serve as a pattern for gene variant testing. In this regard, we have developed a standardized framework and metric for evaluation of uncertain gene variants, with the idea that rather than giving a clinician a 'black box' interpretation of uncertain gene variants, the evidence and decision-making is transparent to clinicians so they can use this in consultation with the patient to make treatment decisions.

It is likely that providing this type of information will impact clinical decision making. While critics may argue that relying solely on a computational framework might 'mislead' clinicians in that we do not have the best evidence (that is, a true known genotype-phenotype correlation), the reality is that clinicians still have to make treatment decisions based on any 'uncertain significance' result. We propose that increasing the transparency of gene test evidence and interpretation would only help the clinician as compared to a situation where results that are on the border of benign and those on the border of pathogenic are treated the same. As Consensus is implemented into a laboratory setting, coordination with a clinical site to test how clinicians use the information would be an important and necessary follow-up study.

The lack of a widely accepted standard for computational predictors in a clinical setting remains a serious obstacle in the diagnostic utility of these algorithms. Gene-specific prediction algorithms have been shown to be an improvement over existing generalized prediction tools, where a larger data set 'n' for training algorithms may not compensate for lower quality of phenotype information. Examples of this gene-disease-specific focus using computational prediction have recently been shown for hypertrophic cardiomyopathy and in the *RET *proto-oncogene [35,44]. We have recently summarized similar efforts in gene-specific prediction for an authoritative 20 gene-disease data set showing similar improved prediction [45]. Focusing prediction algorithms on authoritative and specific gene-disease settings may aid to bridge this acceptance gap and shed additional light on clinical interpretation of uncertain gene variants. With ongoing efforts to amass gene variation in human disease, newly emerging 'authoritative' or 'diagnostic grade' clinically curated gene variant archives should be leveraged for training and testing machine learning classification tools.

Medical geneticists rely on patient history, family segregation, literature review and trusted colleagues to stay informed of the phenotypic consequences of a given gene variant. In addition, well established computer prediction tools are often employed [16,46]. One recent report (Condel) details combining various algorithms into a single score to assess 'deleteriousness' in nonsynonymous (missense) variants [36]. Supporting computational methods may serve to replicate this same mental process of gathering evidence from complementary sources, assessing agreement of the evidence and summarizing this evidence into a clinical context for interpretation of the gene variant finding [47].

For scenarios lacking conventional gene variant evidence, the five specific predictors used for Consensus were carefully chosen due to the varied computational approach of each algorithm. Analysis of variance shows the majority of the weighted average stems from three of the five predictors (PSAAP, MutPred and PMut). This may be indicative of the unique and varied approach of the three predictors. SIFT and PolyPhen were also included in the Consensus score for a 'wisdom of the crowd' historical context due to the fact that many laboratories may already have these prediction algorithms in use.

One limitation of this methodology is the fact that although several popular gene variants collections are ongoing (dbSNP has recently passed the 12 million unique human gene variant milestone), a relatively small number of clinically curated and authoritative gene-disease collections exist as used for diagnostic purposes. Fortunately, this number will likely continue to expand over time, not diminish, as gene-disease associations are better understood and personalized patient treatments advance. Another limitation is that mutation archives often have an unbalanced proportion of disease-causing gene variants, and appropriate machine learning techniques must be used to compensate for uneven training and test data. In addition, a given gene variant may not only result in a missense change as being evaluated here, but may also impact splicing and translation of a gene product and thus be deleterious even when an apparently benign effect is expected.

Perhaps the most important limitation to acknowledge is how can we know whether a prediction for a gene variant of uncertain significance is truly correct? The honest response is likely 'we can not'. While only the passage of time may confirm the accuracy of a computational prediction, an important point not to dismiss is - would this approach (or similar) likely lead to better or worse decision-making by providers? One recent article points to the importance of careful curation in locus-specific databases [48] and these collections should be leveraged for algorithm improvements. There may also be analogous situations in other existing laboratory tests, where, for example, anatomic pathology may yield some ideas that clinicians rely on for decision-making. The pathology report contains all information, not just the 'interpretation'. Importantly, this would imply that more information (not less) is appropriate for clinician decision-making [19,49].

Another key issue is that disease classification of gene variants evolves over time as new knowledge becomes available. We note that this is a problem whether one uses this proposed framework or the status quo system for dealing with gene test results of uncertain significance. At present, there is no way to communicate new variant knowledge effectively between gene test laboratories and clinicians. Thus, a standardized framework would allow for consistent and objective data provenance for longitudinal tracking of both variants and patient results, where notifying interested parties in updated variant classification and disease association would be more feasible. We also note that development of this framework now using monogenic diseases may allow increased understanding that could eventually be applied to multi-gene panel or whole genome approaches.

There may be some perceived liability of a laboratory that would report using this augmented methodology as compared to existing gene test reporting approaches. Although correlation of genotype-phenotype offers therapeutic options that would otherwise remain hidden and may lead to disease-specific, mutation-guided management strategies, appropriate caution is justified when clinicians are asked to trust computational outcomes for determining patient care [50]. On the other hand, when results are reported to clinicians and patients as variants of unknown significance, it may take years for sufficient molecular or family evidence to be confirmed for the laboratory to make a final determination. Interpretation of gene test results that are unclear or uncertain may be troubling for patients, and must have some effect (good or bad) on how clinicians manage these patients [51]. Transparent communication of summarized gene variant evidence and continued interaction between clinicians and laboratorians to refine mutation-specific clinical classification is imperative to optimal patient care. Recent examples of this importance have been detailed in newborn screening and case studies from cardiovascular genetics [52,53].

## Conclusions

The vision of personalized medicine invokes an image of all relevant information being available to clinicians on demand. Proper interpretation of gene test results is one key area in customizing patient therapy [54,55]. Gene variants are currently being identified at a tremendous pace. While many of these sequence changes may be considered as normal population allele variants, some percentage will certainly have disease association. Gene variants may be best leveraged for clinical utility by focusing on specific gene-disease areas. In concert, clinicians and diagnostic laboratories are the best source of authoritative gene variant annotation. Ranking agreement through the use of a weighted Consensus metric of predicted pathogenicity across several complementary algorithms may provide a level of clinical confidence in computational classifiers.

**Figure 5:**
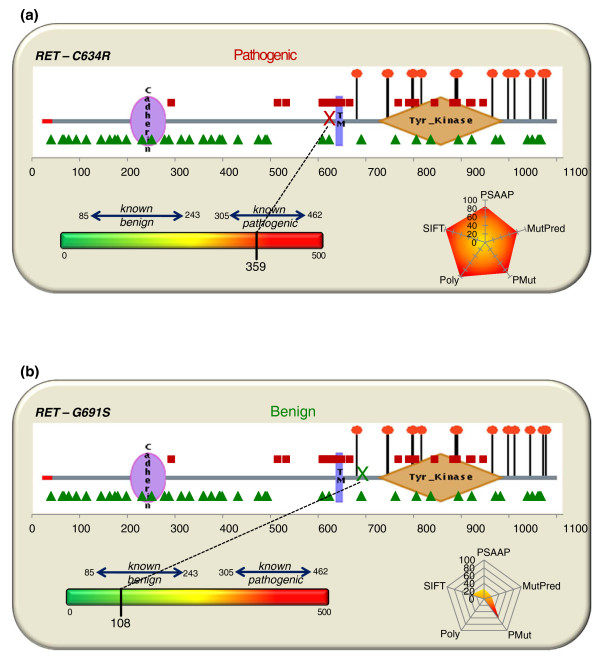
**Protein view with Consensus scoring**. **(a, b) **Proposed visualization of Consensus scoring using known gene variants plotted on the RET_HUMAN (UniProt #P07949) protein (image courtesy of the Human Protein Reference Database [56]) and weighted algorithm output with radar plots to summarize predictor evidence for pathogenic gene variant C634R (a), scoring 359, and benign variant G691S (b) with a Consensus score of 108.

A proposed visual for augmenting the gene test report of an uncertain gene variant using known benign and pathogenic gene variants mapped onto a schematic of the RET protein is displayed in Figure 5. The protein diagram image is courtesy of the Human Protein Reference Database [56]. The variant being evaluated is denoted by 'X' along the length of the protein and Consensus scoring of the variant is detailed using both the reference intervals with colorimetric scale and a radial chart to show the contribution of each predictor. Ongoing efforts include expanding the Consensus scoring framework and phenotype reference intervals to additional genes and diseases. Future efforts will be necessary to incorporate algorithm layers for nucleotide-level prediction and functional protein motifs.

## Abbreviations

AUC: area under the curve; CI: confidence interval; PSAAP: Primary Sequence Amino Acid Properties; SNP: single nucleotide polymorphism.

## Competing interests

The authors declare that they have no competing interests.

## Authors' contributions

DC, EL and MSW assisted in gene variant data collection and helped draft the manuscript. PR, AW and DC participated in the design of the study, performed the statistical analysis and drafted the manuscript. DC, SN, JF and JM conceived of the study, and participated in its design and coordination and helped to draft the manuscript. All authors read and approved the final manuscript for publication.

## Supplementary Material

Additional file 1 **Additional figures**. Figures S1 and S2: test results from representative algorithms such as Scolioscore and FibroTest. Figure S3: analysis of variance explained as determined using principal components. **(A) **Scree plot of descending eigenvalues displaying the five principal components corresponding to the combined predictor algorithms. **(B) **Percent variance explained corresponding to the proportion of cumulative input of five combined predictors. Click here for file

Additional file 2 **Additional tables**. Table S1: five predictor results for benign *RET *gene variants. Table S2: five predictor results for pathogenic *RET *gene variants. Table S3: five predictor results for uncertain *RET *gene variants. Table S4: descriptive statistics, correlation of predictors and significance for *RET *gene variants with known disease association. Table S5: principal components and eigenvalues of predictor scores from *RET *gene variants with known disease association. Click here for file
