# Supplementary material for: Consensus: a framework for evaluation of uncertain gene variants in laboratory test reporting
Source: Genome Med. 2012 May 28;4(5):48. doi: 10.1186/gm347 (PMC3506914; doi:10.1186/gm347)
Supplement: Additional file 1 — Additional figures. Figures S1 and S2: test results from representative algorithms such as Scolioscore and FibroTest. Figure S3: analysis of variance explained as determined using principal components. (A) Scree plot of descending eigenvalues displaying the five principal components corresponding to the combined predictor algorithms. (B) Percent variance explained corresponding to the proportion of cumulative input of five combined predictors. [file gm347-S1.PDF]

Supplemental Figure 1

axial BIOTECH™

ScoliScore™ AIS Prognostic Test

2749 East Parleys Way, Suite 200, Salt Lake City, UT 84109-9921
Toll Free: (877) 294-2598 | Tel: (801) 984-9098 | Fax: (801) 984-9099
www.axialbiotech.com

AIS Prognostic Assay Test Result Form

PATIENT INFORMATION

Patient Name: Doe, Jane Gender: ☐ M ☒ F DOB: 01/01/1995

Medical Record/Patient #: 555-666-7777 Specimen Barcode ID: AXIAL - 00233

Facility Name: Community Medical Indication: Adolescent Idiopathic Scoliosis

Requisition Form #: AXIAL - 00233 Date Received: 01/01/2009 Date Reported: 01/15/2009

Physician Name: Dr. John Smith Additional Recipient: Julie Smith, R.N.

ASSAY DESCRIPTION

The ScoliScore™ AIS Prognostic Test is performed on DNA extracted from saliva, a multiplex PCR reaction is used to determine the genotype for a panel of 53 single nucleotide polymorphisms (SNPs). The ScoliScore™ AIS Progression Score which ranges from 1 – 200 is calculated using marker weighting factors and a simple additive algorithm.

ASSAY RESULTS

Draft

SNP ID #	GENOTYPE	SNP ID #	GENOTYPE	SNP ID #	GENOTYPE	SNP ID #	GENOTYPE	SNP ID #	GENOTYPE
Marker 1	GG	Marker 12	GG	Marker 23	CT	Marker 34	GG	Marker 45	AG
Marker 2	CT	Marker 13	CT	Marker 24	AA	Marker 35	CT	Marker 46	CC
Marker 3	AA	Marker 14	AA	Marker 25	CC	Marker 36	AA	Marker 47	GG
Marker 4	CC	Marker 15	CC	Marker 26	AG	Marker 37	CC	Marker 48	AG
Marker 5	AG	Marker 16	AG	Marker 27	CT	Marker 38	AG	Marker 49	CC
Marker 6	CC	Marker 17	CC	Marker 28	AA	Marker 39	CC	Marker 50	GG
Marker 7	GG	Marker 18	GG	Marker 29	CC	Marker 40	GG	Marker 51	GG
Marker 8	CT	Marker 19	CT	Marker 30	AG	Marker 41	CT	Marker 52	GG
Marker 9	AA	Marker 20	AA	Marker 31	CT	Marker 42	AA	Marker 53	CTv
Marker 10	CC	Marker 21	CC	Marker 32	AA	Marker 43	CC		
Marker 11	CT	Marker 22	AG	Marker 33	CC	Marker 44	AG		

SCOLISCORE™ AIS PROGRESSION SCORE = 190

ASSAY INTERPRETATION

This score indicates this patient is at high risk for progression of a severe curve by or before skeletal maturity. The ScoliScore™ Test should not be used as the sole basis for treatment or disease monitoring decisions, but should be interpreted in conjunction with other diagnostic information for the patient with a diagnosis of AIS. All other available clinical information should be taken into consideration when counseling the AIS patient regarding the risk of curve progression or appropriate treatment. Note: The ScoliScore™ Test is NOT intended for the initial diagnosis of asymptomatic family members or relatives of diagnosed AIS patients. Studies have NOT established the effectiveness of the ScoliScore™ Test other than for Caucasian patients at this time. Therefore, the test results should not be relied on for patients outside this population. Additional studies with other ethnic groups are currently ongoing.

1 ↑ 190 200

ASSAY ACCURACY

Sensitivity: 90% **Specficity:** 88% although rare, genotyping errors can occur due to misincorporation of DNA bases by the enzyme used to perform the test, sample misidentification, sample contamination, or general laboratory errors.

Laboratory Director: Kenneth Ward, M.D. ______________________________

This Laboratory Developed Test was developed and its performance characteristics determined by Axial Biotech Laboratories. The test has not been cleared or approved by the U.S. Food and Drug Administration (FDA). The FDA has determined that such clearance or approval is not necessary for Laboratory Developed Tests. This laboratory is regulated under the Clinical Laboratory Improvement Amendments of 1988 (CLIA) and is qualified to perform high-complexity clinical testing. These results are adjunctive to the ordering physician's diagnosis.

CLIA Number: 46D1077919

©2008, Axial Biotech, Inc. ScoliScore™ is a trademark of DePuy Spine, Inc. | Form #100-290A This test is performed pursuant to an agreement with Roche Molecular Systems, Inc.

Supplemental Figure 2

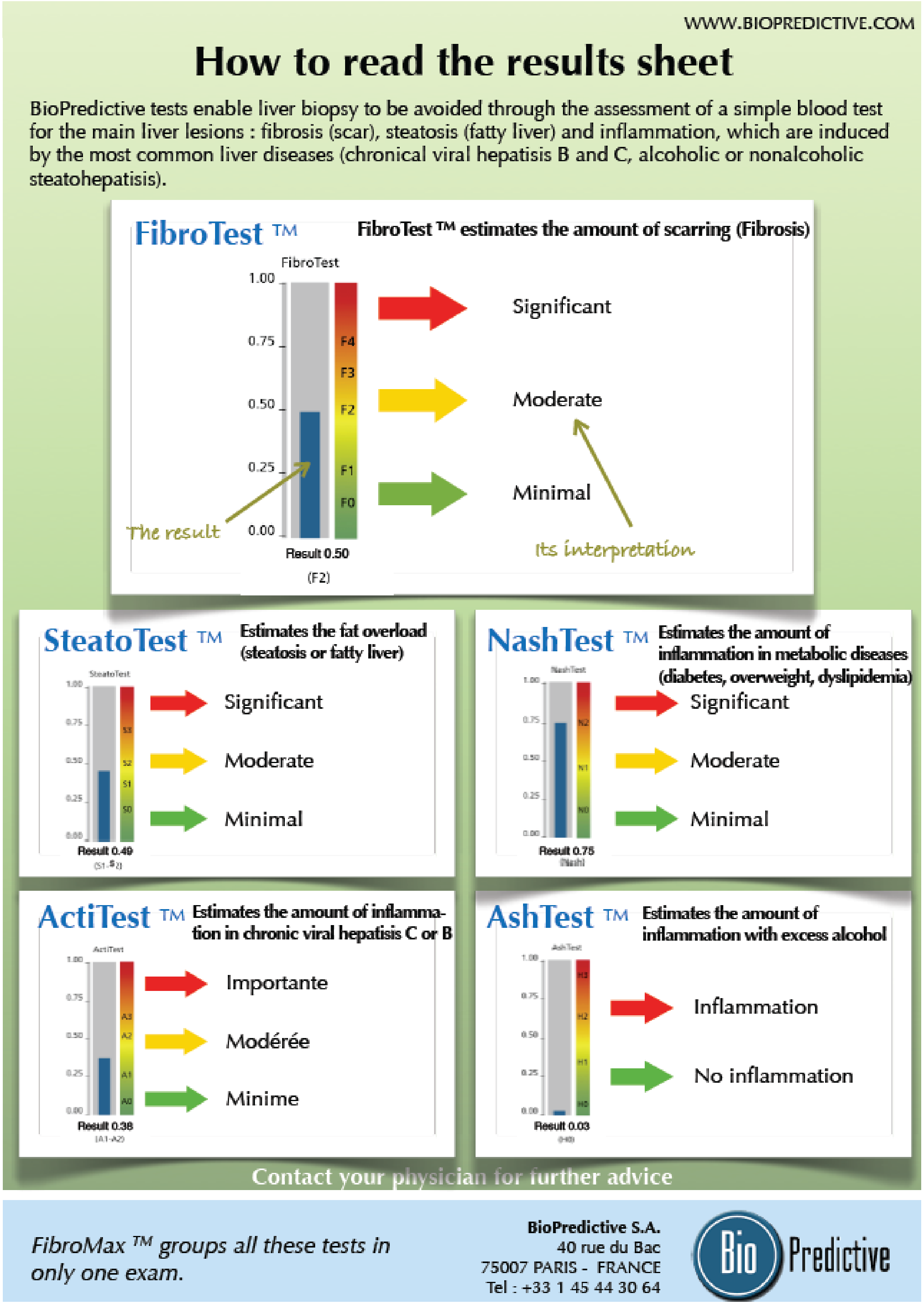

Supplemental Figure 3

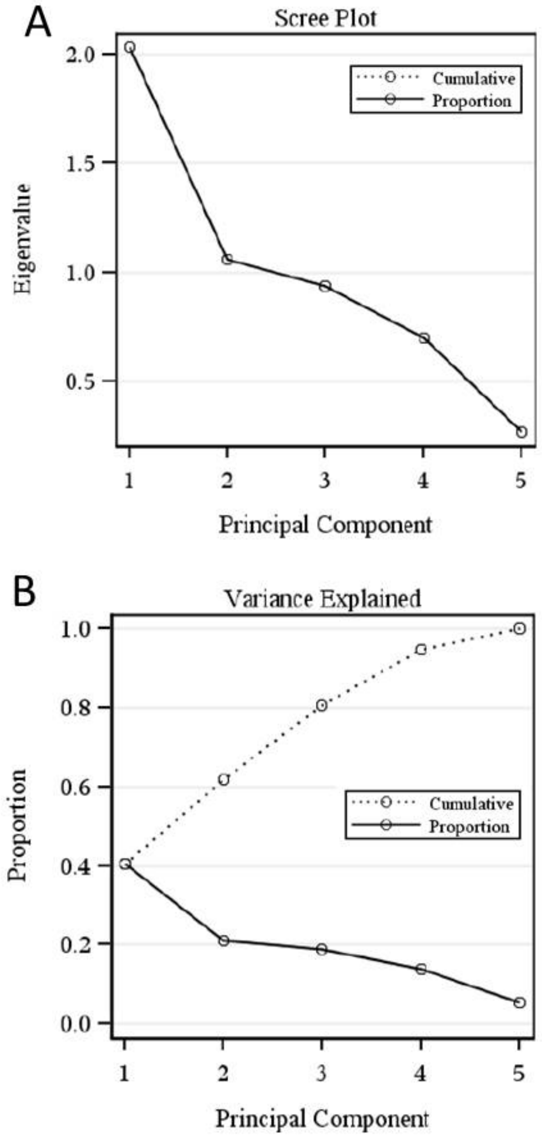

Supplemental Figure 3. Analysis of variance explained as determined using principal components. A) Scree plot of descending eigenvalues displaying the five principal components corresponding to the combined predictor algorithms. B) Percent variance explained corresponding to the proportion of cumulative input of five combined predictors.
