# Supplementary material for: Consensus: a framework for evaluation of uncertain gene variants in laboratory test reporting
Source: Genome Med. 2012 May 28;4(5):48. doi: 10.1186/gm347 (PMC3506914; doi:10.1186/gm347)
Supplement: Additional file 2 — Additional tables. Table S1: five predictor results for benign RET gene variants. Table S2: five predictor results for pathogenic RET gene variants. Table S3: five predictor results for uncertain RET gene variants. Table S4: descriptive statistics, correlation of predictors and significance for RET gene variants with known disease association. Table S5: principal components and eigenvalues of predictor scores from RET gene variants with known disease association. [file gm347-S2.DOC]

**Supplemental Table 1. Five predictor results for benign *RET* gene variants.**

Variant^a^ PSAPP^b^ MutPred^c^ PMUT^d^ PolyPhen^e^ SIFT^f^

A432E 0.12 0.00 0.63 0.79 1.00

C609W 0.24 0.91 0.89 0.99 0.50

D489N 0.97 0.00 0.24 1.00 0.20

E251K 0.16 0.00 0.63 1.00 0.64

E623K 0.14 0.51 0.83 1.00 0.80

E762Q 0.23 0.68 0.04 1.00 0.09

F1112Y 0.23 0.00 0.03 1.00 0.37

F174S 0.19 0.00 0.11 1.00 0.09

F393L 0.17 0.00 0.39 1.00 0.20

G691S 0.26 0.20 0.66 0.00 0.66

G894S 0.58 0.99 0.77 1.00 0.50

G93S 0.11 0.00 0.51 1.00 0.00

L1061P 0.09 0.75 0.65 1.00 0.10

L40P 0.19 0.00 0.08 1.00 0.00

M1064T 0.14 0.58 0.70 0.75 0.13

N359K 0.16 0.00 0.64 1.00 0.25

N394H 0.25 0.00 0.40 1.00 0.01

N394K 0.12 0.00 0.52 1.00 0.01

P1039Q 0.07 0.72 0.66 0.98 0.30

P1049L 0.09 0.57 0.84 1.00 0.20

P1067S 0.13 0.29 0.79 1.00 0.21

P198T 0.21 0.00 0.62 1.00 0.01

P20L 0.23 0.00 0.27 1.00 0.16

P399L 0.03 0.00 0.72 1.00 0.03

P64L 0.16 0.00 0.17 1.00 0.01

R114H 0.18 0.00 0.61 1.00 0.16

R163Q 0.03 0.00 0.53 0.00 0.25

R180P 0.25 0.00 0.68 1.00 0.24

R231H 0.10 0.00 0.48 0.98 0.56

R287Q 0.12 0.00 0.47 0.75 0.02

R313Q 0.25 0.00 0.57 0.37 0.03

R330Q 0.10 0.00 0.57 0.96 0.44

R360W 0.17 0.00 0.94 0.65 0.00

R475Q 0.08 0.00 0.71 0.79 0.29

R67H 0.15 0.00 0.25 1.00 0.55

R77C 0.11 0.00 0.26 1.00 0.06

R972G 0.52 0.91 0.71 1.00 0.10

R982C 0.16 0.89 0.65 1.00 0.70

S493E 0.07 0.00 0.66 1.00 0.97

S690P 0.62 0.53 0.46 1.00 0.14

S836Y 0.13 0.46 0.78 1.00 0.77

S922Y 0.61 0.92 0.77 0.82 0.80

T278N 0.12 0.00 0.22 1.00 0.29

V145G 0.12 0.00 0.31 1.00 0.00

V376A 0.07 0.13 0.19 0.04 0.60

Y806C 0.67 0.90 0.91 0.43 0.81

^a^ RET_HUMAN (UniProt #P07949) used as reference amino acid sequence.

^b^ Primary Sequence Amino Acid Properties (PSAAP) algorithm, gene-specific trained.

^c^ Analyzed with default settings at http://mutdb.org/mutpred.

^d^ Analyzed with default settings at http://mmb.pcb.ub.es/PMut.

^e^ Analyzed with default settings at http://genetics.bwh.harvard.edu/pph.

^f^ Analyzed with default settings at http://sift.jcvi.org.

**Supplemental Table 2. Five predictor results for pathogenic *RET* gene variants.**

Variant^a^ PSAPP^b^ MutPred^c^ PMUT^d^ PolyPhen^e^ SIFT^f^

A640G 0.82 0.58 0.13 0.86 0.34

A883F 0.95 0.91 0.64 0.99 0.00

C515S 0.91 0.00 0.83 0.02 0.19

C609F 0.77 0.79 0.84 0.99 0.00

C609G 0.94 0.89 0.88 0.99 0.00

C609R 0.91 0.87 0.93 0.01 0.00

C609S 0.87 0.90 0.61 0.97 0.00

C609Y 0.85 0.90 0.98 0.97 0.00

C611F 0.92 0.93 0.73 1.00 0.00

C611G 0.86 0.92 0.80 1.00 0.00

C611R 0.77 0.92 0.86 1.00 0.00

C611S 0.77 0.90 0.42 0.00 0.00

C611W 0.78 0.94 0.80 0.31 0.00

C611Y 0.74 0.96 0.96 0.29 0.00

C618F 0.79 0.90 0.86 0.20 0.58

C618G 0.88 0.88 0.89 0.97 0.41

C618R 0.97 0.85 0.94 0.99 0.28

C618S 0.97 0.85 0.80 0.94 0.50

C618W 0.89 0.77 0.90 1.00 0.13

C618Y 0.75 0.89 0.98 0.98 0.84

C620F 0.81 0.86 0.81 1.00 0.23

C620G 0.87 0.83 0.70 1.00 0.46

C620R 0.79 0.75 0.88 1.00 0.69

C620S 0.91 0.81 0.70 1.00 0.74

C620W 0.85 0.84 0.81 0.00 0.06

C620Y 0.80 0.84 0.96 0.99 0.00

C630F 0.78 0.76 0.61 0.00 0.06

C630R 0.83 0.70 0.79 1.00 0.84

C630S 0.90 0.73 0.42 1.00 0.34

C630Y 0.87 0.75 0.94 1.00 1.00

C634F 0.85 0.83 0.75 1.00 0.04

C634G 0.82 0.78 0.84 1.00 0.02

C634L 0.77 0.69 0.39 1.00 0.80

C634R 0.85 0.79 0.88 1.00 0.02

C634S 0.87 0.82 0.43 1.00 0.04

C634W 0.89 0.83 0.81 1.00 0.01

C634Y 0.90 0.81 0.97 1.00 0.13

D631Y 0.87 0.69 0.68 1.00 0.02

E768D 0.47 0.56 0.16 1.00 0.13

G533C 0.78 0.00 0.79 1.00 0.00

K666E 0.85 0.51 0.24 1.00 0.31

L790F 0.97 0.80 0.12 1.00 0.01

M918T 0.41 0.54 0.79 1.00 0.00

R844L 0.39 0.84 0.83 1.00 0.10

S649L 0.87 0.66 0.46 0.99 0.65

S891A 0.78 0.67 0.08 1.00 0.31

S922F 0.42 0.89 0.68 1.00 0.00

T946M 0.89 0.76 0.78 0.89 0.60

V292M 0.95 0.50 0.96 1.00 0.14

V804L 0.84 0.73 0.17 1.00 0.03

V804M 0.90 0.77 0.11 1.00 0.09

^a^ RET_HUMAN (UniProt #P07949) used as reference amino acid sequence.

^b^ Primary Sequence Amino Acid Properties (PSAAP) algorithm, gene-specific trained.

^c^ Analyzed with default settings at http://mutdb.org/mutpred.

^d^ Analyzed with default settings at http://mmb.pcb.ub.es/PMut.

^e^ Analyzed with default settings at http://genetics.bwh.harvard.edu/pph.

^f^ Analyzed with default settings at http://sift.jcvi.org.

**Supplemental Table 3. Five predictor results for uncertain *RET* gene variants.**

Variant^a^ PSAPP^b^ MutPred^c^ PMUT^d^ PolyPhen^e^ SIFT^f^

G321R 0.41 N/A 0.74 0.00 0.50

A510V 0.11 N/A 0.25 0.00 0.08

E511K 0.17 N/A 0.78 0.01 0.24

C531R 0.60 N/A 0.83 1.00 0.28

G533S 0.40 N/A 0.14 0.99 0.00

R600Q 1.00 N/A 0.41 0.00 0.27

K603Q 0.40 N/A 0.45 0.00 0.47

Y606C 0.38 0.54 0.91 0.90 0.23

C609S 0.92 0.90 0.61 1.00 0.00

C611S 0.77 0.90 0.42 1.00 0.00

C630S 0.79 0.73 0.42 1.00 0.34

D631N 0.89 0.61 0.18 0.05 0.19

D631A 0.53 0.65 0.75 0.98 0.01

D631G 0.31 0.62 0.51 0.93 0.02

D631V 0.77 0.60 0.64 0.98 0.00

D631E 0.14 0.39 0.24 0.85 0.07

E632K 0.40 0.63 0.16 0.13 0.19

R635G 0.41 0.71 0.76 1.00 0.10

A640G 0.22 0.58 0.13 0.00 0.50

A641S 0.26 0.59 0.02 0.29 0.09

V648I 0.30 0.58 0.10 0.00 0.75

S649L 0.46 0.66 0.46 1.00 0.32

H665Q 0.30 0.52 0.58 1.00 0.57

K666N 0.07 0.49 0.16 0.99 0.33

R770Q 1.00 0.44 0.49 1.00 0.08

N777S 0.27 0.65 0.35 0.28 0.64

V778I 0.30 0.52 0.02 0.00 0.02

Y791N 0.78 0.89 0.86 0.99 0.00

E805K 0.68 0.88 0.66 1.00 0.00

E818K 0.40 0.51 0.16 0.38 0.19

S819I 0.73 0.77 0.48 1.00 0.00

R833C 0.70 0.59 0.91 1.00 0.00

P841L 0.54 0.60 0.81 1.00 0.67

E843D 0.81 0.70 0.04 0.00 0.12

R844W 0.84 0.77 0.99 1.00 0.00

R844Q 1.00 0.64 0.65 1.00 0.06

R844L 0.83 0.84 0.83 1.00 0.02

M848T 0.62 0.67 0.80 1.00 0.08

I852M 0.84 0.74 0.07 0.99 0.04

L881V 0.60 0.81 0.11 0.96 0.00

R886W 0.74 0.78 0.98 1.00 0.00

S904C 0.82 0.72 0.61 0.98 0.01

S904F 0.75 0.65 0.83 1.00 0.00

K907E 0.60 0.71 0.27 0.99 0.00

K907M 0.58 0.64 0.30 0.99 0.00

R912Q 1.00 0.94 0.55 1.00 0.00

^a^ RET_HUMAN (UniProt #P07949) used as reference amino acid sequence.

^b^ Primary Sequence Amino Acid Properties (PSAAP) algorithm, gene-specific trained.

^c^ Analyzed with default settings at http://mutdb.org/mutpred.

^d^ Analyzed with default settings at http://mmb.pcb.ub.es/PMut.

^e^ Analyzed with default settings at http://genetics.bwh.harvard.edu/pph.

^f^ Analyzed with default settings at http://sift.jcvi.org.

**Supplemental Table 4. Descriptive statistics, correlation of predictors and significance for *RET* gene variants with known disease association.**

***Simple Statistics***

Variable N Mean Std Dev Median Minimum Maximum

MutPred 97 0.5072 0.3881 0.6900 0.0000 0.9900

PMut 97 0.6070 0.2729 0.6798 0.0342 0.9809

Poly 97 0.8537 0.3112 0.9990 0.0000 1.0000

PSAAP 97 0.5336 0.3446 0.6700 0.0300 0.9700

SIFT 97 0.2401 0.2927 0.1300 0.0000 1.0000

***Spearman Correlation Coefficients***

MutPred PMUT PolyPhen PSAAP

MutPred **

PMut 0.541 **

Poly -0.204 -0.289 **

PSAAP 0.562 0.250 -0.134 **

SIFT -0.296 -0.118 0.001 -0.118

***P-value of correlation***

MutPred PMUT PolyPhen PSAAP

**

<.0001 **

0.0452 0.0041 **

<.0001 0.0134 0.1898 **

0.0033 0.2496 0.9953 0.2497

**Supplemental Table 5. Principal components and eigenvalues of predictor scores from *RET* gene variants with known disease association.**

Eigenvalues of the Correlation Matrix

Eigenvalue Difference Proportion Cumulative

1 2.03136792 0.97261793 0.4063 0.4063

2 1.05874999 0.11844368 0.2117 0.6180

3 0.94030630 0.23882133 0.1881 0.8061

4 0.70148497 0.43339416 0.1403 0.9464

5 0.26809082 0.0536 1.0000

Eigenvectors

Prin1 Prin2 Prin3 Prin4 Prin5

MutPred 0.622645 0.141730 0.219197 -.017230 -.737483

PMut 0.464901 0.357804 -.064845 0.754559 0.286843

Poly -.273897 0.401935 0.773679 -0.400519 0.066595

PSAAP 0.563184 0.245172 0.247588 -.440005 0.606474

1 – SIFT 0.063118 0.793869 -.536535 0.276267 0.039931
